# Supplementary figures and images for: A Recovery-Oriented Program for People with Bipolar Disorder through Virtual Reality-Based Cognitive Remediation: Results of a Feasibility Randomized Clinical Trial
Source: J Clin Med. 2023 Mar 9;12(6):2142. doi: 10.3390/jcm12062142 (PMC10056011; doi:10.3390/jcm12062142)

Figures S 1,2,3,4,5. CEREBRUM scenarios

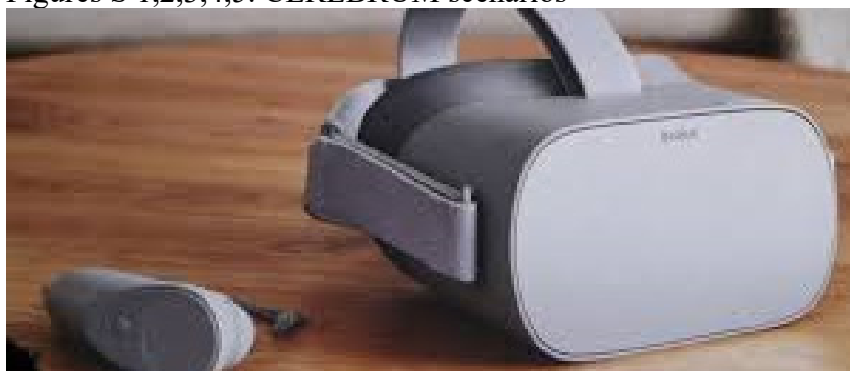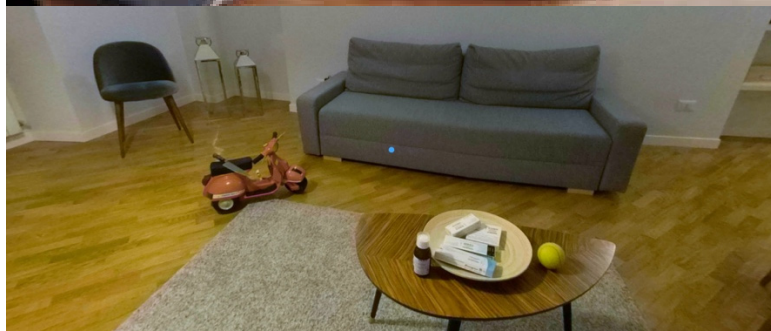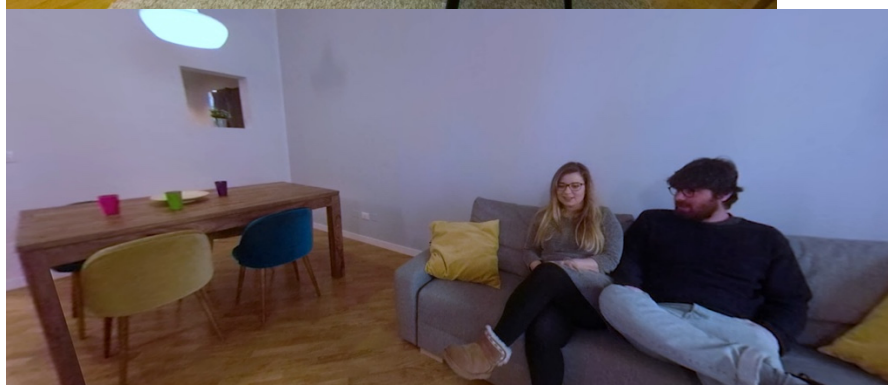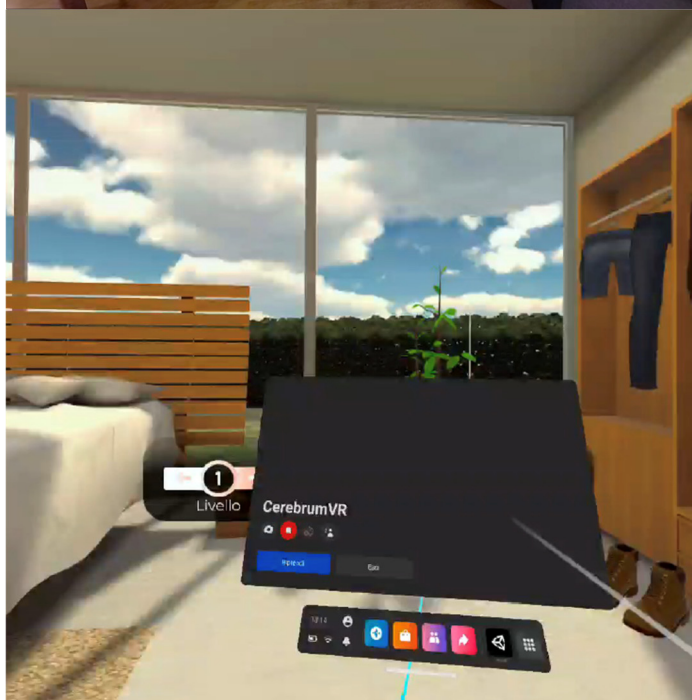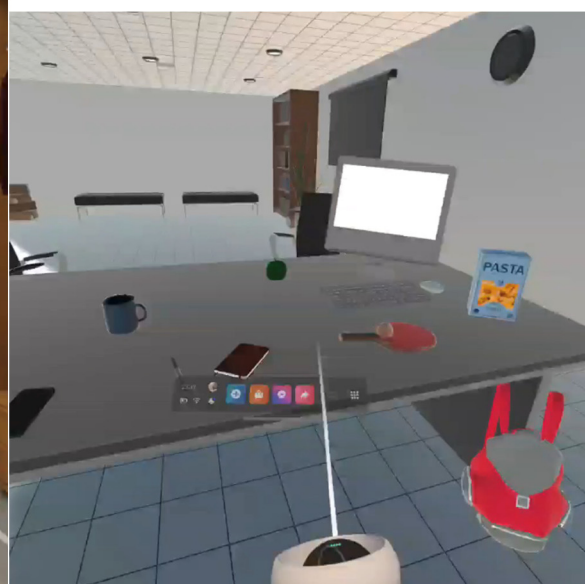

Supplement: Supplementary file 1 [file jcm-12-02142-s001.zip › jcm-2181812-supplementary.pdf]
